# Research progress on risk factors of recurrence of benign paroxysmal positional vertigo

**DOI:** 10.3389/fneur.2025.1735898

**Published:** 2026-01-12

**Authors:** Jianhua Liu, Jiang Liu, Yongchuan Dai, Feng He, Hongxiang Zhai

**Affiliations:** 1 Department of Otorhinolaryngology, Xinghua People’s Hospital Affiliated to Yangzhou University, Xinghua, Jiangsu, China; 2 Department of Oncology, Xinghua People’s Hospital Affiliated to Yangzhou University, Xinghua, Jiangsu, China

**Keywords:** benign paroxysmal positional vertigo, bone mineral density, recurrence, risk factors, thyroid function, vascular metabolic diseases, vestibular function, vitamin D deficiency

## Abstract

Benign paroxysmal positional vertigo (BPPV) is a prevalent disorder affecting the peripheral vestibular system. Although repositioning maneuvers can effectively alleviate symptoms in the majority of patients, the recurrence rate remains notably high, which has a significant impact on the quality of life of affected individuals. Consequently, it is essential to investigate the factors that contribute to the recurrence of BPPV. Current studies suggest that BPPV recurrence is associated with an array of factors, including metabolic abnormalities, endocrine disorders, vascular-metabolic diseases, prior head trauma, as well as gender and age-related factors. Further, vestibular dysfunction, genetic predispositions, and immunological factors also play a role. This paper aims to provide a comprehensive analysis of the mechanisms by which these factors influence BPPV recurrence. By synthesizing the most recent clinical research and meta-analyses, this study elucidates the clinical significance of BPPV. It also addresses relevant prevention and intervention strategies to equip clinicians to offer effective treatments and enhance long-term outcomes for patients experiencing BPPV.

## 1 Introduction

BPPV is recognized as the most prevalent peripheral vestibular disorder. The condition is characterized by the dislodgement of otoconia, small calcium carbonate crystals located in the inner ear, from the utricle in response to changes in head position. These crystals subsequently migrate into the semicircular canals, where they can move freely (1, 2), resulting in an increased sensitivity of the labyrinth to gravitational forces and eliciting brief episodes of rotational vertigo. The pathogenesis of BPPV is multifaceted, encompassing alterations in various structures and functions within the inner ear. According to the diagnostic criteria established by the Bárány Society in 2015 (3), the clinical manifestations of BPPV include vertigo and nystagmus, which are triggered by specific positional changes. While these criteria constitute a milestone in the diagnosis and management of BPPV, areas of uncertainty and ambiguity remain in clinical practice, particularly concerning the diverse clinical subtypes and underlying pathophysiological mechanisms (3).

The diagnosis of Benign Paroxysmal Positional Vertigo (BPPV) typically involves the observation of characteristic nystagmus during positional tests, such as the Dix–Hallpike maneuver. The features and duration of the nystagmus observed can be instrumental for clinicians in identifying specific inner ear involvement. Recent advancements in clinical research related to BPPV have provided greater insights into its various subtypes, including Posterior Semicircular Canal BPPV with cupulolithiasis (PSC-BPPV-cu) and PSC-BPPV with canalolithiasis (PSC-BPPV-sa), particularly concerning their clinical manifestations and treatment responses (4). Although current treatment modalities for BPPV demonstrate a high degree of success, the elevated recurrence rate poses a significant clinical challenge. Research indicates that recurrence rates vary considerably among studies, ranging from 13.3 to 65%, contingent upon the duration of follow-up and specific patient characteristics (5). This issue has garnered substantial attention within the academic community.

Recent investigations have indicated that the recurrence of Benign Paroxysmal Positional Vertigo (BPPV) may be associated with a variety of factors, including age, sex, comorbidities (such as hypertension, hyperlipidemia, diabetes, and vitamin D deficiency), and lifestyle choices (5, 6). Evidence suggests that females exhibit a higher prevalence of both the initial onset and recurrence of BPPV (6), while elderly patients demonstrate a significantly elevated rate of recurrence (7, 8). Furthermore, vitamin D deficiency has been closely linked to an increased risk of BPPV recurrence (9). Additionally, the recurrence of BPPV is closely related to the interactions among metabolic, immune, neural, and vascular systems; however, the precise underlying mechanisms remain partially understood. This review systematically summarizes the factors contributing to BPPV recurrence, explores potential mechanisms and their clinical implications, and offers new insights aimed at enhancing the prognosis for patients affected by BPPV.

## 2 Risk factors of recurrence of BVVP

### 2.1 Vitamin D deficiency

#### 2.1.1 Correlation between vitamin D deficiency and vestibular function

Vitamin D is recognized for its significant role in vestibular function. Otoliths, which are critical components of the vestibular system located in the inner ear, enable the body to perceive its position and movement. Prior studies have indicated that deficiencies in vitamin D may lead to impaired mineralization of otoliths, thereby increasing the risk of otolith detachment—a principal mechanism underlying benign paroxysmal positional vertigo (BPPV) (10). Furthermore, individuals with vitamin D deficiency exhibit a higher prevalence of abnormalities in tests assessing vestibular evoked myogenic potential (VEMP) and caloric responses (11). This suggests that vitamin D may influence vestibular function by impacting the stability and mineralization processes of otoliths. The hypothesis also posits that vitamin D deficiency may disrupt normal vestibular function through mechanisms such as impaired neural signaling, osteoporotic changes within the otic capsule, and alterations in otolith particles (12). Moreover, among the elderly population, vitamin D deficiency is significantly correlated with an increased incidence of vestibular disorders, which may contribute to heightened risks of falls and dizziness (13). A study involving 138 patients diagnosed with BPPV demonstrated that individuals with normal vitamin D levels were significantly less likely to present vestibular abnormalities compared to those exhibiting vitamin D deficiency (11). This finding indicates a substantial difference in vestibular function associated with varying levels of vitamin D status.

#### 2.1.2 The correlation between vitamin D deficiency and recurrence of BPPV

Vitamin D deficiency has been identified as a significant factor associated with the recurrence of benign paroxysmal positional vertigo (BPPV). Research indicates that individuals with insufficient levels of vitamin D exhibit a markedly higher recurrence rate of BPPV compared to those with adequate vitamin D status. A specific study demonstrated that vitamin D concentrations were significantly lower in patients with BPPV in contrast to a control group, with individuals experiencing recurrence displaying substantially reduced vitamin D levels compared to those in the non-recurrence cohort. This finding suggests that vitamin D deficiency may play a critical role in both the initiation and recurrence of BPPV (14). Furthermore, a multivariate analysis revealed that patients with elevated serum vitamin D levels were less likely to experience recurrent episodes of BPPV, with an odds ratio (OR) of 0.83 (95% confidence interval [CI] 0.76–0.90, *p* < 0.001). These findings underscore the correlation between vitamin D deficiency and an increased risk of BPPV recurrence, reinforcing the hypothesis concerning the involvement of vitamin D in the pathogenesis of BPPV (15, 16). It is proposed that vitamin D may impact the development and recurrence of BPPV through its effects on calcium metabolism and bone mineralization within the inner ear.

#### 2.1.3 Clinical application and limitations of vitamin D supplementation therapy

Vitamin D deficiency is closely associated with the development and recurrence of benign paroxysmal positional vertigo (BPPV). Research indicates that active supplementation of vitamin D significantly reduces both the recurrence rate and the severity of this condition (17, 18). Multiple controlled studies and meta-analyses have demonstrated that patients with BPPV who receive vitamin D supplementation experience a significantly lower recurrence rate compared to untreated control groups (10, 16, 19). Other meta-analyses have also indicated that vitamin D supplementation notably decreases the annual recurrence rate of BPPV, especially in patients with vitamin D levels below 20 ng/mL (14, 17). Despite the positive effects observed with vitamin D supplementation in treating BPPV, some limitations exist. First, the diversity in study designs complicates the comparison and synthesis of results, with variations in definitions and criteria—such as the threshold for vitamin D deficiency—potentially leading to inconsistent conclusions (10). Additionally, some studies involve relatively small sample sizes, which may impact the reproducibility and reliability of findings (20, 21). Moreover, evidence suggests that vitamin D supplementation should be tailored to individual patient conditions, taking into account baseline health status and other comorbidities, in order to develop personalized treatment plans (20). Consequently, treatment regimens for vitamin D supplementation—including dosage, frequency, and duration—vary significantly across studies, necessitating careful evaluation of their safety and efficacy. Therefore, there is a pressing need to establish unified guidelines to optimize treatment approaches informed by clinical experience (Table 1).

**Table 1:** Summary of risk factors for recurrence of benign paroxysmal positional vertigo (BPPV).

Risk factor	Mechanism	Key findings	OR/RR/HR (95% CI)	Sample size	References
Vitamin D deficiency	Impaired otoconia mineralization, altered calcium metabolism	Lower Vit D levels in recurrent BPPV vs. non-recurrent group (*p* < 0.001); OR = 0.83 (0.76–0.90) for recurrence prevention with Vit D supplementation	RR = 0.41 (*p* < 0.01)	138	(16)
Osteoporosis/BMD reduction	Weakened otoconia adhesion, microarchitectural deterioration	T-score < −1.0 in 73% BPPV patients vs. 35% controls (*p* = 0.002); 67.3% recurrence rate in postmenopausal women with osteoporosis	HR = 1.62 (1.21–2.16)	65	(29)
Thyroid dysfunction	Autoimmune-mediated inner ear damage, altered vestibular neurotransmission	neurotransmission24.4% recurrence rate in hypothyroid BPPV vs. 14.4% in euthyroid group (*p* = 0.0006); Elevated TPO-Ab/TG-Ab associated with recurrence (*p* < 0.05)	OR = 3.27 (1.45–7.38)	797	(67)
Vascular-metabolic disorders	Microangiopathy-induced ischemia, oxidative stress	53.48% recurrence rate in diabetics; Hypertension associated with 2.3-fold increased recurrence risk (*p* < 0.001)	RR = 1.89 (1.32–2.71)	1,289	(5)
Genetic predisposition	Familial aggregation of otoconia structural anomalies	Insertion variant in PCDHGA10 gene linked to familial recurrent BPPV; Adjusted OR = 14.10 for family history	OR = 14.10 (*p* < 0.001)	214	(82)
Female gender	Estrogen-mediated otoconia stabilization, hormonal fluctuation	In both age groups (45–65 years old and >65 years old), patients who took estrogen for menopausal syndromes had a significantly lower incidence of BPPV	aHR = 0.01, (0.06–0.23), *p* < 0.001	7200	(50)
Age factor	Migraine,Vascular disorders of the central nervous system, Heart disorders	Mean age of the first BPPV was 52.8 ± 14.5 years; there were females (76.9%), while (67.3%) of patients presented recurrences within two years of follow-up	aHR = 0.1161 (0.07–0.15)	3042	(53)
Head trauma history	Otoconia dislodgement, labyrinthine concussion	22.1% 5-year recurrence rate in trauma-associated BPPV vs. 13.3% in idiopathic cases (*p* = 0.003)	RR = 1.68 (1.12–2.51)	548	(47)
Vestibular dysfunction	Persistent canalithiasis, reduced VEMP symmetry	Abnormal oVEMP asymmetry in 57.7% recurrent BPPV vs. 38% first-episode cases;		76	(61)
Psychological factors	Anxiety-induced autonomic dysfunction, increased symptom perception	Anxiety scores correlated with 2.1-fold increased recurrence risk (*p* = 0.008); 38% mediating effect of depression on vertigo severity	OR = 2.024 (1.57–2.608)	1056	(84)

### 2.2 Decreased bone mineral density and abnormal bone metabolism

#### 2.2.1 Relationship between osteoporosis and otolith mineralization

Osteoporosis is a systemic condition characterized by reduced bone density and the deterioration of bone microarchitecture. Individuals with osteoporosis frequently exhibit vitamin D deficiency, which can adversely affect the mineralization of otoconia. Vitamin D plays a crucial role in facilitating calcium absorption and bone mineralization; thus, a deficiency in this vitamin can lead to inadequate mineralization of otoconia, consequently elevating the risk of benign paroxysmal positional vertigo (BPPV). Research indicates that the structure of otoconia in patients with osteoporosis may be more fragile due to alterations in the bone matrix and mineral loss, rendering the otoconia more susceptible to detachment and subsequently triggering episodes of BPPV (22–24).

Bone metabolic disorders signify disruptions in the processes involved in bone formation and remodeling, resulting in diminished bone quality. Recent studies have established a notable correlation between bone metabolic dysregulation and BPPV. Specifically, in osteoporotic individuals, aberrant bone metabolism may impair both the mineralization and adhesive characteristics of otoconia, increasing their likelihood of dislodgement during changes in head position and thus precipitating episodes of vertigo (25, 26). Therefore, interventions aimed at addressing bone metabolic disorders—such as calcium and vitamin D supplementation, along with the administration of anti-osteoporotic medications—may enhance bone density and the mineralization status of otoconia, ultimately reducing the occurrence of BPPV attacks.

#### 2.2.2 The correlation between bone mineral density and BPPV recurrence

The association between decreased bone mineral density and the recurrence of benign paroxysmal positional vertigo (BPPV) has garnered increasing attention within the medical community. Evidence suggests that individuals with reduced bone density experience a significantly higher recurrence rate of BPPV compared to those with normal bone density (24, 27). In particular, postmenopausal women with BPPV typically exhibit lower bone density than their healthy counterparts, rendering them more vulnerable to vertigo symptoms during changes in head position (28). A study involving 65 postmenopausal female patients demonstrated that bone density measurements indicated significantly lower T-scores in those diagnosed with BPPV when compared to normal women. This finding implies that reduced bone density may be a significant factor contributing to the elevated recurrence rate of BPPV (29). Furthermore, a systematic review underscores that the relationship between osteoporosis and BPPV is especially pronounced in patients aged 50 and above (30).

Despite the suggestion of a correlation between bone density and the recurrence of BPPV, some studies within the existing literature present conflicting results. This disparity highlights the necessity for further large-scale prospective studies to elucidate the specific role of bone metabolism in the recurrence of BPPV (31, 32).

### 2.3 Related factors of vascular metabolic diseases

#### 2.3.1 The effect of hypertension, diabetes, and hyperlipidemia on the recurrence of BPPV

When analyzing the factors associated with the recurrence of benign paroxysmal positional vertigo (BPPV), it is essential to consider the impact of metabolic disorders such as hypertension, diabetes, and hyperlipidemia. These vascular conditions can impede the blood supply to the vestibular system, thereby adversely affecting the metabolism and function of the otoliths. Recent studies indicate that the prevalence of hypertension and hyperlipidemia among BPPV patients is significantly elevated compared to the general population. This disparity may be attributed to microvascular damage linked to these conditions, which can result in the displacement or dysfunction of otoliths (5). Moreover, there is evidence that fluctuations in blood pressure are closely associated with the blood supply to the inner ear. Specifically, individuals with hypertension may experience insufficient inner ear perfusion due to postural changes, thereby increasing the likelihood of BPPV episodes (33). The recurrence rate of BPPV in diabetic patients may be as high as 53.48% (5), suggesting that diabetic neuropathy may further impair the regulatory mechanisms of the vestibular system (6). Consequently, metabolic disorders such as hypertension, diabetes, and hyperlipidemia significantly contribute to the increased recurrence rate of BPPV by disrupting otolith metabolism and the blood supply to the inner ear. It is imperative for clinicians to prioritize the management of these comorbidities when assessing patients with BPPV, as this approach can mitigate the risk of recurrence, enhance overall prognosis, and improve the quality of life for affected individuals (34–36).

#### 2.3.2 Pathophysiological machine

Microangiopathy refers to the impairment or dysfunction of microvessels, including capillaries and arterioles, which play a crucial role in various neurological disorders. In patients diagnosed with benign paroxysmal positional vertigo (BPPV), microangiopathy may compromise blood flow to the inner ear, consequently leading to vestibular dysfunction. The labyrinthine artery serves as the sole source of blood supply to the inner ear, branching into fine vessels devoid of collateral circulation. Insufficient perfusion can adversely affect the normal functionality of the cochlea and vestibular organs, resulting in symptoms such as vertigo and imbalance (37, 38). When microangiopathy is associated with chronic conditions such as diabetes, hypertension, and hyperlipidemia, these underlying diseases can exacerbate hypoxia within the inner ear, further impairing the sensitivity and operational capacity of vestibular receptors (39). A study has indicated that patients with BPPV demonstrated symptoms associated with microcirculatory disturbances, implying that microangiopathy may significantly contribute to the pathogenesis of BPPV (40). Additionally, metabolic disorders can modify the inner ear environment by influencing inflammatory responses and increasing oxidative stress levels. Oxidative stress is recognized as a critical factor in the damage and dysfunction of inner ear cells, and metabolic disturbances can elevate oxidative stress, thereby aggravating inner ear injury (41, 42). In summary, a thorough investigation into the relationship between microangiopathy, metabolic disorders, and vestibular dysfunction is imperative for elucidating the pathophysiological mechanisms underlying BPPV and for developing more effective clinical intervention strategies.

### 2.4 Previous head trauma

#### 2.4.1 Head trauma as a risk factor for recurrence

Head trauma is recognized as a significant risk factor for the recurrence of benign paroxysmal positional vertigo (BPPV). Mechanical injury resulting from head trauma can lead to the detachment of otoconia, ultimately causing vestibular dysfunction. Numerous studies have demonstrated that patients with a history of head trauma exhibit a markedly increased rate of BPPV recurrence. For instance, one study analyzed a cohort of 4,291 patients who developed non-positional vertigo following head injury and identified 244 cases diagnosed with non-positional peripheral vestibular disorders, where recurrent vestibular pathology was the most prevalent cause (43). This finding underscores the substantial role of head trauma in precipitating vestibular dysfunction. The underlying mechanism for the onset of BPPV following head trauma may be associated with the displacement of otoconia as well as structural damage to the inner ear. Upon head impact, otoconia within the inner ear may detach from their usual position and migrate into the semicircular canals, leading to symptoms of vertigo during positional changes of the head (44). Furthermore, head trauma can induce hemodynamic changes in the inner ear, adversely affecting its function and heightening the risk of BPPV recurrence (45). It is noteworthy that even mild head trauma, such as minor concussions sustained during sports or traffic accidents, has been closely linked to the occurrence of BPPV (46).

In a prospective study, it was determined that 22.1% of 548 BPPV patients experienced a recurrence within 5 years, with head trauma identified as a significant risk factor for such recurrence (47). Another study corroborated that head trauma is not only associated with the initial manifestation of BPPV but is also significantly correlated with an increased frequency of recurrent episodes. This information is vital for clinicians involved in the management of BPPV patients (48). Consequently, for patients with BPPV and a history of head trauma, it is imperative to pay close attention to their risk of recurrence and to implement timely intervention strategies designed to mitigate this recurrence rate.

### 2.5 Gender and age factors

#### 2.5.1 Women are associated with the risk of recurrence

The elevated recurrence rate of benign paroxysmal positional vertigo (BPPV) among women compared to men has attracted considerable attention from the research community. Several studies indicate that women experience a significant decline in estrogen levels during middle age and menopause. Preliminary research suggests that estrogen may play a critical role in the stability and functionality of otoliths by regulating calcium metabolism within the inner ear (49, 50). This regulatory function may be a contributing factor to the onset and recurrence of BPPV. Moreover, the role of estrogen extends beyond calcium regulation; it is thought to modulate various metabolic processes in inner ear cells, which may influence the formation and function of otoliths within the vestibular system (12). Such modulation may disrupt the mineralization process of the otoliths, affecting their quality and quantity, and increasing their susceptibility to detachment and movement into the semicircular canals, thereby precipitating episodes of vertigo. In addition, certain studies have reported a correlation between hormonal fluctuations and the frequency of BPPV episodes within the context of the menstrual cycle or pregnancy, further emphasizing the significant influence of estrogen on BPPV occurrence (51). Conversely, other research indicates that appropriate hormone replacement therapy may mitigate the incidence and recurrence of BPPV by enhancing bone metabolism and promoting the health of otoliths (49, 52). The evidence regarding the mechanistic role of hormone replacement therapy in BPPV is indeed derived primarily from preclinical animal models, while supportive human data remains observational. Nonetheless, further exploration through larger-scale clinical trials and long-term observational studies is essential to substantiate the validity of these hypotheses.

#### 2.5.2 The relationship between age and recurrence rate

The relationship between age and the recurrence rate of benign paroxysmal positional vertigo (BPPV) represents a significant area of investigation in medical research. A prior study has demonstrated that the mean age of elderly patients at the initial onset of BPPV is 52.8 years, accompanied by a notably high recurrence rate of 67.3% (53). Furthermore, the incidence of osteoporosis markedly increases among elderly individuals, and this condition is closely associated with vitamin D deficiency. Vitamin D is crucial not only for maintaining bone health but also for facilitating proper vestibular function within the inner ear (54). Research indicates that vitamin D deficiency may contribute to both the occurrence and recurrence of BPPV, particularly within the elderly population. In this demographic, vitamin D supplementation has been shown to significantly alleviate recurrence rates (32), underscoring how age-related physiological changes can heighten susceptibility to BPPV. Additionally, the degeneration of vestibular function associated with aging is a critical factor that influences the recurrence of BPPV. As individuals advance in age, the vestibular system—including the cochlea, vestibular nerve, and relevant neural pathways—undergoes degeneration. This decline in functionality impairs the ability of older adults to adapt to changes in head position, thus increasing their vulnerability to vertiginous episodes (55). This finding further substantiates the connection between aging and BPPV recurrence.

Elderly patients frequently exhibit multiple comorbidities, including hypertension, diabetes, and cardiovascular diseases. These underlying conditions can adversely affect the micro-environment of the inner ear, consequently elevating the risk of BPPV recurrence (56). Empirical studies reveal that elderly individuals with hypertension and diabetes experience a significantly higher recurrence rate of BPPV compared to those without these conditions (57–59). Therefore, it is imperative to conduct systematic assessments and implement targeted interventions for elderly patients, especially for those presenting with metabolic abnormalities or osteoporosis.

### 2.6 Vestibular dysfunction and neural mechanism

#### 2.6.1 Abnormal vestibular evoked myoelectric response and recurrence

Vestibular Evoked Myogenic Potentials (VEMPs) constitute a vital neurophysiological assessment employed to evaluate the functionality of the vestibular system. In the context of benign paroxysmal positional vertigo (BPPV), abnormal findings observed in ocular VEMP (oVEMP) and cervical VEMP (cVEMP) are frequently associated with vestibular dysfunction (60). Research indicates that patients with recurrent BPPV demonstrate a significantly greater amplitude asymmetry in oVEMP compared to individuals experiencing their first episode. This observation implies that vestibular impairment may persist even during asymptomatic periods (61), which could contribute to the recurrence of BPPV. Furthermore, vitamin D deficiency has been identified as a significant factor influencing vestibular dysfunction. Empirical studies suggest that individuals with suboptimal vitamin D levels exhibit a higher frequency of abnormalities in vestibular function assessments, including both oVEMP and cVEMP, in contrast to those with sufficient vitamin D levels. Enhancing vitamin D status may also mitigate the risk of recurrence associated with BPPV (11). Consequently, abnormal VEMP responses are considered indicators of vestibular dysfunction. Emerging evidence suggests a potential association between these abnormalities and a higher likelihood of BPPV recurrence, positing that persistent subclinical vestibular impairment may contribute to recurrence. However, further studies are needed to establish its value as a reliable clinical predictor.

#### 2.6.2 Neuropathogenesis

Vestibular nerve conduction abnormalities are recognized as a significant factor contributing to the recurrence of benign paroxysmal positional vertigo (BPPV). The pathogenesis of BPPV involves dysfunction within the vestibular system of the inner ear, with particular emphasis on the semicircular canals. Research indicates that patients diagnosed with BPPV exhibit impaired vestibular nerve function, which enhances their sensitivity to alterations in head position. This increased sensitivity renders individuals more susceptible to the onset of vertigo symptoms during routine daily activities (62). Moreover, abnormal vestibular nerve conduction may interact with other inner ear pathologies, such as otolith displacement or inner ear infections, potentially exacerbating symptoms and contributing to recurrence (63, 64). Further studies suggest that individuals with a familial history of BPPV may possess a more vulnerable vestibular nerve function, thereby increasing susceptibility to recurrence following head trauma or other triggers (65). This insight opens new avenues for clinical intervention. It has also been observed that following multiple episodes of BPPV, patients may undergo adaptive changes in their vestibular system. Such pathological neuroplasticity may lead to abnormal responses to positional stimuli, consequently heightening the risk of recurrence (66). Additionally, research has shown that vitamin D deficiency may affect neuroplasticity and, consequently, the recurrence rate of BPPV (14). Therefore, clinicians may consider vitamin D supplementation as a potential strategy to enhance neuroplasticity and reduce the likelihood of BPPV recurrence.

### 2.7 Association between thyroid disorders, autoimmunity, inflammation, and recurrence of benign paroxysmal positional vertigo (BPPV)

#### 2.7.1 Thyroid disorders and BPPV recurrence

A significant association exists between thyroid dysfunction and BPPV recurrence. In a study of 797 primary BPPV patients, the recurrent BPPV (R-BPPV) group had a higher prevalence of hypothyroidism (24.4%, 61/250) under long-term hormone replacement therapy (HRT) compared to the non-recurrent BPPV (NR-BPPV) group (14.4%, 79/547; *p* = 0.0006) (67). Moreover, Hashimoto’s thyroiditis (HT) has been found to be closely linked to BPPV recurrence, with correlation analyses yielding significant results (*p* < 0.0001) (67) Elevated serum levels of thyroid peroxidase antibody (TPO-Ab; *p* = 0.0117) and thyroglobulin antibody (TG-Ab; *p* = 0.0025) further confirmed the link between autoimmune thyroid responses and BPPV recurrence (67). A large-scale analysis of 434,552 BPPV patients reinforced this, showing abnormal thyroid hormone levels are associated with both BPPV onset and recurrence (45), underscoring the need to assess thyroid function in clinical risk evaluation.

#### 2.7.2 Autoimmune diseases and recurrence risk

Beyond thyroid disorders, various autoimmune conditions increase BPPV recurrence risk. Patients with systemic lupus erythematosus, multiple sclerosis, or HTerosis, or HT with systemic lupus erythematosus, multiple sclerosis, or HTevels are associated with both BPPV onset and controls (58, 68–70). Notably, Approximately 67.3% of BPPV patients experience recurrence within 2 years, closely tied to their immune-inflammatory status (58). Middle-aged women show a pronounced correlation between anti-thyroid antibodies and recurrence, suggesting autoimmune mechanisms contribute to BPPV pathogenesis in this demographic (71) Immune-mediated inflammation plays a central role: dysregulated immune responses release cytokines and inflammatory mediators, damaging inner ear structures (e.g., sensory/supporting cells of the vestibular system) and exacerbating vertigo (58). Inner ear inflammation can damage the cochlea and vestibular organs, triggering positional vertigo—especially in the elderly, where immune responses may accelerate vestibular degeneration (72, 73).

#### 2.7.3 Autoimmune mechanisms in BPPV pathogenesis

Autoimmune mechanisms are increasingly implicated in otolaryngological disorders, including BPPV. Autoimmune encephalitis often presents with systemic positional vertigo, initially misdiagnosed as BPPV, where autoantibodies (e.g., GAD65) disrupt vestibular signaling, causing balance impairment (45). The hypothesis of immune-mediated vestibular injury gains support from evidence that immune responses induce anatomical/functional changes in the vestibular system, affecting auditory and balance functions (72). For example, inflammation in autoimmune inner ear diseases damages vestibular structures, leading to recurrent vertigo similar to BPPV (72).

#### 2.7.4 Inflammatory markers and systemic inflammation

Inflammatory factors, including thyroid-related markers and systemic inflammation, significantly influence the recurrence of Benign Paroxysmal Positional Vertigo (BPPV). A study comprising 250 patients diagnosed with Recurrent BPPV (R-BPPV) and 547 patients with Non-Recurrent BPPV (NR-BPPV) demonstrated markedly elevated levels of thyroid antibodies in the recurrent group, indicating a potential link to autoimmune responses (67). Furthermore, systemic inflammatory conditions such as hypertension and diabetes correlate with an increased incidence of BPPV recurrence. Notably, patients with hypertensive BPPV exhibit a substantially higher recurrence rate, likely due to altered systemic inflammatory responses that may compromise the stability of the inner ear (63).

#### 2.7.5 Potential of immunomodulatory therapies

Immune regulation is critical for addressing BPPV recurrence, particularly in comorbidities like type 2 diabetes mellitus (T2DM). T2DM is a recognized risk factor, but genomic studies remain limited. Differential gene expression and weighted gene co-expression network analyses have identified shared genes between BPPV recurrence and T2DM, offering insights into immunomodulatory targets (6). Using the CIBERSORT algorithm to map immune cell infiltration, researchers found upregulated key genes in blood samples from BPPV patients with comorbid T2DM and recurrent episodes, suggesting these genes may serve as predictive biomarkers for recurrence risk (6).

These findings collectively highlight the interplay of thyroid dysfunction, autoimmunity, and inflammation in BPPV recurrence. This evidence supports a targeted clinical approach for patients at higher risk of recurrence. For individuals with recurrent or atypical BPPV, a focused assessment is warranted. This could include: 1. A detailed medical history screening for symptoms or a known diagnosis of thyroid disease, autoimmune conditions (e.g., lupus, rheumatoid arthritis), or metabolic disorders (e.g., diabetes). 2. Selective laboratory testing based on clinical suspicion, which may include thyroid-stimulating hormone (TSH), anti-thyroid antibodies (TPO-Ab, TG-Ab), vitamin D level, and basic inflammatory markers (e.g., ESR, CRP). The goal of this targeted evaluation is to identify and address modifiable systemic conditions that may contribute to vestibular vulnerability, thereby personalizing management beyond canalith repositioning procedures alone.

### 2.8 Lifestyle and environmental factors

#### 2.8.1 Sports and occupation related factors

High-intensity exercise has a notable impact on the onset and recurrence of benign paroxysmal positional vertigo (BPPV). Studies suggest that vigorous physical activity may cause microtrauma to the inner ear structures, thereby increasing the risk of BPPV. In a case report, an association between football (soccer) and BPPV was suggested, with head trauma from the sport proposed as a potential precipitating factor (44). While headers or impacts in contact sports represent a plausible mechanistic risk factor for inner ear microtrauma, this specific link requires further systematic investigation. Furthermore, a prospective case–control study involving 393 patients diagnosed with vertigo revealed that occupational exposure to noise and mechanical vibration considerably increases the risk of experiencing vertigo. The prevalence of exposure to noise and vibration was notably greater among vertigo patients than in the control group, a trend observed in both male and female participants. This finding highlights occupational exposure as a critical risk factor for BPPV. Furthermore, sustained poor posture, such as the forward-head posture common during prolonged computer use, has been investigated as a potential contributor to dizziness. Research in patients with cervical degenerative changes has associated such postures with alterations in cervical biomechanics and vertebral artery hemodynamics (68, 69). Research has demonstrated that sustained static postures may also compromise cervical blood circulation and neural transmission, thereby reducing the adaptability of the vestibular system and increasing the likelihood of BPPV recurrence (70). It has been hypothesized that these mechanisms could, theoretically, influence vestibular function or compensation; however, a direct causal link to BPPV incidence or recurrence remains speculative and warrants targeted research.

#### 2.8.2 Seasonal and environmental impacts

The connection between seasonal and environmental factors and the recurrence of benign paroxysmal positional vertigo (BPPV) has gained significant attention in the clinical community. A systematic review and meta-analysis indicate an inverse correlation between the incidence of BPPV and serum vitamin D levels in the Northern Hemisphere, suggesting that lower vitamin D levels are associated with a higher risk of BPPV onset (71). This finding implies that exposure to sunlight and seasonal variations may indirectly influence the recurrence of BPPV by affecting vitamin D synthesis. Moreover, climate change and shifts in weather patterns may also impact the recurrence of BPPV. Research conducted by Paolo Mariani revealed a significantly higher proportion of BPPV cases during the winter and autumn months compared to the spring and summer seasons (74). Additionally, studies by Shu et al. provided evidence that serum vitamin D levels were markedly lower in patients diagnosed with BPPV during winter than in those from the summer and autumn cohorts (75). These observations suggest that seasonal declines in vitamin D may be related to increased rates of BPPV recurrence. Further studies by Saeed and Omari, Mariani et al., and Cao et al. identified a significant negative correlation between temperature and the occurrence of BPPV (74, 76, 77). It is also noteworthy that individuals tend to adopt a more sedentary lifestyle during colder months, which may lead to reduced outdoor activity. This lack of physical activity could contribute to bone demineralization and osteoporosis, potentially increasing the risk of BPPV. Currently, the existing literature does not indicate any correlation between humidity and BPPV (76, 78, 79). The complex interplay of these factors complicates the understanding of how seasonal influences affect the recurrence of BPPV.

#### 2.8.3 Living habits intervention

Lifestyle modifications are critical for reducing the recurrence rate of benign paroxysmal positional vertigo (BPPV). Research suggests that certain changes in daily habits can significantly influence both the occurrence and recurrence risk of BPPV. A meta-analysis reveals that individuals who engage in vitamin D supplementation exhibit a significantly lower recurrence rate compared to the non-supplementation group (RR = 0.41, *p* < 0.01). This finding underscores the potential importance of vitamin D in preventing the recurrence of BPPV (16). In addition, investigations suggest that patients who consistently participate in balance training and physical exercise demonstrate a markedly reduced recurrence rate of BPPV compared to sedentary counterparts (80). Individuals should adopt a dietary regimen characterized by low sodium and sugar, while emphasizing high-fiber foods. Diets high in salt, sugar, and fat are associated with the development of hypertension and diabetes, which are recognized risk factors for the recurrence of BPPV (32). Furthermore, a prospective study analyzing clinical data from patients diagnosed with BPPV indicated that those receiving multiple treatment modalities exhibited a notably higher recurrence rate. The analysis also disclosed that individuals diagnosed with gout present an elevated risk of recurrence (48). Lastly, psychological well-being is a pivotal factor in the recurrence of BPPV. In another study, no risk factors were identified that were associated with the occurrence of BPPV (81). In summary, by enhancing lifestyle factors through vitamin D supplementation, increased physical activity, optimized dietary habits, and a focus on mental health, patients may improve their overall health and significantly reduce the risk of BPPV recurrence.

### 2.9 Other potential influencing factors and future research directions

#### 2.9.1 Hereditary factors and family history

The mechanisms underlying the recurrence of benign paroxysmal positional vertigo (BPPV) remain incompletely understood; however, an increasing body of research suggests that genetic factors may significantly contribute to certain cases. Recent studies employing whole-exome sequencing have identified an insertion variant in the PCDHGA10 gene that may be associated with familial recurrent BPPV. This investigation demonstrated that the variant has the potential to facilitate the formation of large aggregates of the mutant protein in samples obtained from individuals with BPPV. Furthermore, carriers of this variant exhibit a tendency to develop symptoms earlier than non-carriers (65). Additionally, findings from another study indicate that individuals with a familial history of BPPV are at a markedly increased risk of recurrence, with an adjusted odds ratio reaching as high as 14.10 (82). This observation suggests that genetic background may play a pivotal role in determining an individual’s susceptibility to BPPV, thereby underscoring the significant influence of genetic factors in the pathogenesis of this disorder. As the understanding of the genetic basis of BPPV advances, it may facilitate the development of more effective prevention and treatment strategies aimed at mitigating the recurrence of this condition.

#### 2.9.2 Psychological factors and anxiety and depression

A growing body of research highlights the significant impact of psychological factors on patients diagnosed with benign paroxysmal positional vertigo (BPPV), with particular emphasis on anxiety and depression. A multicenter prospective cohort study demonstrated a notable correlation between anxiety levels and the recurrence of BPPV, revealing that patients exhibiting anxiety are at a substantially heightened risk of recurrence when compared to their non-anxious counterparts (83). In a separate study involving 1,056 patients with BPPV, the quality of sleep was found to be negatively correlated with the severity of vertigo. In this context, anxiety and depression were identified as mediators in the relationship, accounting for 28.5 and 38% of the mediating effects on the severity of vertigo, respectively (84). Furthermore, a large-scale investigation indicated that psychological interventions significantly reduced patients’ scores on the Dizziness Handicap Inventory (DHI), suggesting an improvement in the perception of vertigo-related disability and an enhancement in overall quality of life. This improvement was accompanied by marked reductions in both anxiety and depression scores, as well as diminished sensitivity to and fear of vertigo episodes (85).

#### 2.9.3 Emerging biomarkers and diagnostic techniques

In recent years, biomarkers have demonstrated considerable potential in the investigation of benign paroxysmal positional vertigo (BPPV). Notably, serum Otolin-1 levels have emerged as a promising biomarker for the recurrence of BPPV. Research indicates that Otolin-1 levels are significantly elevated in patients with BPPV compared to healthy control groups, thereby providing foundational support for its role as a predictive marker for both the onset and recurrence of the condition (86, 87). Moreover, a study concentrating on newly diagnosed BPPV patients identified a significant correlation between increased Otolin-1 levels and the risk of recurrence. This finding suggests that Otolin-1 could be clinically advantageous in evaluating the likelihood of recurrence in affected individuals (88). In summary, the incorporation of emerging biomarkers, such as Otolin-1, holds the potential to enhance the precision of predicting BPPV recurrence. This advancement may lead to more effective monitoring and intervention strategies within clinical practice.

In the discipline of imaging and functional testing technologies, contemporary high-resolution computed tomography (CT) and magnetic resonance imaging (MRI) are essential for excluding alternative potential causes of symptoms. These advanced imaging modalities also enhance clinicians’ understanding of the anatomical structures and pathological alterations within the inner ear of patients diagnosed with Benign Paroxysmal Positional Vertigo (BPPV) (89, 90). Furthermore, functional assessment instruments, such as video nystagmography (VNG) and the head impulse test (HIT), play a valuable role in objectively documenting nystagmus characteristics, helping to differentiate between BPPV subtypes, and assessing vestibular function to exclude other peripheral or central pathologies that may mimic or coexist with BPPV (91, 92).

The integration of machine learning and artificial intelligence has significantly enhanced the diagnostic capabilities for Benign Paroxysmal Positional Vertigo (BPPV). Recent studies demonstrate that machine learning models can effectively identify features associated with BPPV through the analysis of clinical data, resulting in improved diagnostic sensitivity and specificity (93). Furthermore, the employment of explainable artificial intelligence (XAI) technology facilitates a deeper understanding and increased confidence among healthcare professionals regarding the predictions generated by these models, thereby providing substantial support for clinical decision-making processes (93). In conclusion, advanced imaging technologies and innovative functional testing methodologies exhibit considerable potential for the diagnosis and prediction of BPPV recurrence, thereby offering more precise guidance for patient management and treatment strategies.

#### 2.9.4 Construction of multi-factor risk prediction model

The recurrent vertigo symptoms associated with benign paroxysmal positional vertigo (BPPV) can profoundly affect patients’ quality of life. Therefore, developing an effective prediction model to assess the risk of recurrence represents a significant and valuable clinical goal. By integrating clinical and laboratory indicators, healthcare providers can more accurately identify high-risk patients, thereby facilitating the development of individualized treatment and prevention strategies. Recent studies have identified various clinical factors—specifically hypertension, diabetes, migraines, and vitamin D levels—as significant risk factors for the recurrence of BPPV (94, 95). These factors can be assessed through LASSO regression analysis and multivariate logistic regression analysis, allowing for the construction of a robust prediction model with substantial predictive capability.

A multicenter study employed a forward selection method that incorporated clinical histories, laboratory findings, and patient lifestyle factors to develop a tool known as the “Risk Prediction Model.” The evaluation of this model was conducted using receiver operating characteristic (ROC) curves, the Hosmer–Lemeshow goodness-of-fit test, and decision curve analysis. The findings indicated that the area under the curve (AUC) exceeded 0.7 in both the training and validation cohorts, demonstrating favorable discriminative ability and clinical applicability (94). The establishment of this model not only provides a scientific basis for the early identification of high-risk patients but also supports the formulation of effective preventive strategies.

## 3 Conclusion

The recurrence of Benign Paroxysmal Positional Vertigo (BPPV) presents a complex clinical issue driven by the interaction of multiple factors and mechanisms. A comprehensive review of the existing literature indicates that BPPV recurrence should not be viewed as a mere isolated physiological event; instead, it results from a multifaceted interplay among metabolic, endocrine, vascular, immunological, and neurological components. Current research identifies several well-established risk factors associated with BPPV recurrence, including vitamin D deficiency, diminished bone mineral density, thyroid dysfunction, and vascular-metabolic disorders. These factors may contribute to a heightened risk of BPPV recurrence by modifying the physiological conditions within the inner ear or adversely affecting the overall health status of the individual.

Moreover, factors such as prior head trauma, variations in gender and age, vestibular dysfunction, and immune status play significant roles in the recurrence of Benign Paroxysmal Positional Vertigo (BPPV). These diverse elements emphasize that BPPV recurrence constitutes a complex health issue, for which a singular treatment approach frequently produces suboptimal outcomes. Consequently, clinical practice should prioritize a thorough assessment of these multifactorial aspects to formulate individualized prevention and treatment strategies.

Current research has yet to yield conclusive results; however, it is reasonable to propose that lifestyle factors and psychological conditions may influence the recurrence of Benign Paroxysmal Positional Vertigo (BPPV). It is imperative for future studies to conduct a more in-depth exploration of these areas in order to establish comprehensive guidelines for clinical practice.

Given the complexities involved in managing recurrent Benign Paroxysmal Positional Vertigo (BPPV), the adoption of precision medicine is crucial. It is recommended that clinicians implement individualized intervention strategies that thoroughly consider the unique characteristics and diverse risk factors associated with each patient’s BPPV management. Furthermore, future research should prioritize large-scale, multi-center studies with long-term follow-up, aimed at enhancing risk prediction models for recurrence.
